# A novel intensity-based multi-level classification approach for coronary plaque characterization in intravascular ultrasound images

**DOI:** 10.1186/s12938-018-0586-1

**Published:** 2018-11-06

**Authors:** Ga Young Kim, Ju Hwan Lee, Yoo Na Hwang, Sung Min Kim

**Affiliations:** 1 Department of Medical Biotechnology, Dongguk University-Bio Medi Campus, 32, Dongguk-ro, Ilsandong-gu, Goyang, Gyeonggi-do 10326 Republic of Korea; 2 Department of Medical Devices Industry, 26, Pil-dong 3-ga, Jung-gu, Seoul, 04620 Republic of Korea

## Abstract

**Background:**

Intravascular ultrasound (IVUS) is a commonly used diagnostic imaging method for coronary artery disease. Virtual histology (VH) characterizes the plaque components into fibrous tissue (FT), fibro-fatty tissue (FFT), necrotic core (NC), or dense calcium (DC). However, VH can obtain only a single-frame image in one cardiac cycle, and specific software is needed to obtain the radio frequency data. This study proposed a novel intensity-based multi-level classification model for plaque characterization.

**Methods:**

The plaque-containing regions between the intima and the media-adventitia were segmented manually for all IVUS frames. A total of 54 features including first order statistics, grey level co-occurrence matrix, Law’s energy measures, extended grey level run length matrix, intensity, and local binary pattern were estimated from the plaque-containing regions. After feature extraction, optimal features were selected using principle component analysis (PCA), and these were utilized as the input for the classification models. Plaque components were classified into FT, FFT, NC, or DC using an intensity-based multi-level classification model consisting of three different nets. Net 1 differentiated low-intensity components into FT/FFT and NC/DC groups. Then, net 2 subsequently divided FT/FFT into FT or FFT, whereas the remainder and high-intensity components were classified into NC or DC via net 3. To improve classification accuracy, each net utilized three different input features obtained by PCA. Classification performance was evaluated in terms of sensitivity, specificity, accuracy, and receiver operating characteristic curve.

**Results:**

Quantitative results indicated that the proposed method showed significantly high classification accuracy for all tissue types. The classifiers had classification accuracies of 85.1%, 71.9%, and 77.2%, respectively, and the areas under the curve were 0.845, 0.704, and 0.783. In particular, the proposed method achieved relatively high sensitivity (82.0%) and specificity (87.1%) for differentiating between the FT/FFT and NC/DC groups.

**Conclusions:**

These results confirmed the clinical applicability of the proposed approach for IVUS-based tissue characterization.

## Background

Intravascular ultrasound (IVUS) is a commonly used diagnostic imaging method for coronary artery disease. It takes cross sectional images of the arteries in real time by using catheter with a ultrasound probe and provides diverse information that includes lumen size, plaque rupture, and plaque components. This information is clinically important to determine how to treat a lesion before angioplasty, because different treatment should be apply to each patient according to form of lesion. It also can be used to observe the prognosis after treatment. Additionally, it is useful for early diagnosis of a vulnerable plaque that may cause a stroke or heart attack.

In general, the components of a coronary plaque are manually analysed using visual interpretation method based on grey scale IUVS images. However, each component of plaque shows complicated pattern, which, if not properly recognized, may lead to misdiagnosis of coronary artery disease [1]. Also, diagnostic accuracy mainly depends on the experience of the individual reviewing the images. Therefore, many studies have been automatically classify the plaque components to improve diagnostic results. Among the various methods, virtual histogram (VH), which uses radio frequency (RF) signal data, is regarded as the gold standard in diagnosing coronary artery disease.

VH characterizes the plaque components into fibrous tissue (FT), fibro-fatty tissue (FFT), necrotic core (NC), or dense calcium (DC) based on a combination of information that includes envelope amplitude and underlying frequency content of the RF signal [2]. This information is provided as a colour map, and it can be applied to the diagnosis of various coronary artery diseases that involve a thin-cap fibroatheroma. VH showed high clinical effectiveness for the classification of plaque components, and it has been verified in many previous studies [3, 4]. However, VH showed two main limitations. First, VH can acquire only a single-frame image in one cardiac cycle, because it uses electrocardiogram-gated acquisition [5]. This limits the number of images that can be taken as well as the longitudinal resolution. Also, specific software is needed to obtain the RF signal data [1]. Therefore, it is difficult to apply the VH into the existing equipment.

This study proposed a novel intensity-based multi-level classification model to classify the components of coronary plaque. The proposed method extracts six texture feature sets from the plaque region of an IVUS image. These features were optimized using principle component analysis (PCA). Then, the proposed classification model characterizes plaque components into FT, FFT, NC, or DC.

Main contribution of this study is to improve the classification ability of plaque components in IVUS image by using the new concept of a model, intensity-based multi-level classifier. In addition, extensive features were analysed and optimal feature set was selected among these features. Proposed approach achieved significant classification results to differentiate plaque components. The rest of this paper is laid out as follows. “Methods” section describes the methods, which consist of image acquisition, feature extraction, feature selection, classification, and performance evaluation. Then, “Results” section conveys the experimental results of the proposed method, and “Discussion” section provides the discussion. Finally, “Conclusions” section presents the conclusion of this study.

## Methods

### Image acquisition

This study acquired sequential IVUS images from 11 coronary artery disease patients. It was performed with a 20 MHz 2.9 F phased-array transducer catheter (Eagle Eye, Volcano Corp., Rancho Cordova, California), and grey scale IVUS wasacquired at a constant speed of 0.5 mm/s using a pullback device. The IVUS images consist of 252 frames of 400 × 400 images. The regions of plaque between the intima and media-adventitia were manually segmented by an expert to obtain information regarding the plaque. Segmented images include 1,230,159 pixels that consist of FT, FFT, NC, and DC tissue. This study was approved by Institutional Review Board of Ulsan University Hospital. Written informed consent was obtained from all patients.

### Feature extraction

Feature extraction is the important process of analysing the texture data in images and obtaining meaningful information for the diagnosis of diseases. To obtain sufficient information of pixel, each feature was extracted from a 5 × 5 window region. However, a 3 × 3 window was used to establish a local binary pattern (LBP) in order to analyse the feature information based on neighbour pixels adjacent to the central pixel. Table 1 shows first order statistics (FOS), grey level co-occurrence matrix (GLCM), Law’s energy measures (LEM), extended grey level run length matrix (GLRLM), intensity, and LBP features that were extracted from each mask region.

**Table 1:** Total feature set obtained in the process of feature extraction

Feature set	Feature	Feature set	Feature
FOS	Mean Variance Standard deviation Kurtosis Skewness	LEM	MSS S5S5 MSS R5S5/S5R5 MSS R5R5 MAS E5L5/L5E5 MAS S5L5/L5S5 MAS R5L5/L5R5 MAS E5E5 MAS S5E5/E5S5 MAS R5E5/E5R5 MAS S5S5 MAS R5S5/S5R5 MAS R5R5
GLCM	Autocorrelation Contrast Cluster prominence Cluster shade Dissimilarity Energy Entropy Homogeneity Maximum probability Variance Sum average Sum variance Sum entropy Difference variance Difference entropy Information measure of correlation Normalized inverse difference moment		
		Intensity	Intensity
		GLRLM	Short run emphasis Long run emphasis Grey level nonuniformity Run length nonuniformity Run percentage Low grey level run emphasis High grey level run emphasis Short run low grey level run emphasis Short run high grey level run emphasis Long run low grey level run emphasis Long run high grey level run emphasis
LEM	MSS E5L5/L5E5 MSS S5L5/L5S5 MSS R5L5/L5R5 MSS E5E5 MSS S5E5/E5S5 MSS R5E5/E5R5		
		LBP	Basic LBP Uniform LBP

#### First order statistics

FOS [6] analyzes an original image based on the gray-scale value of each pixel. Unlike second order statistics, it extracts features without considering the relationship between pixels. This study extracted 5 features, mean, variance, standard deviation, kurtosis, and skewness, from 5 × 5 window region. Each feature was calculated using Eqs. (1–5).

1 $$Mean = \frac{1}{I \times J}\sum\limits_{i = 1}^{I - 1} {\sum\limits_{j = 1}^{J - 1} {G(i,j)} }$$

2 $$Variance = \frac{1}{I \times J}\sum\limits_{i = 1}^{I - 1} {\sum\limits_{j = 1}^{J - 1} {\left( {G(i,j) - Mean} \right)^{2} } }$$

3 $$Standard\begin{array}{*{20}c} {} \\ \end{array} deviation = \sqrt {\frac{1}{I \times J}\sum\limits_{i = 1}^{I - 1} {\sum\limits_{j = 1}^{J - 1} {\left( {G(i,j) - Mean} \right)^{2} } } }$$

4 $$Kurtosis = \left[ {\frac{1}{I \times J}\sum\limits_{i = 1}^{I - 1} {\sum\limits_{j = 1}^{J - 1} {\,\left( {\frac{G(i,j) - Mean}{{\sqrt {Variance} }}} \right)}^{4} } } \right] - 3$$

5 $$Skewness = \frac{1}{I \times J}\sum\limits_{i = 1}^{I - 1} {\sum\limits_{j = 1}^{J - 1} {\,\left( {\frac{G(i,j) - Mean}{{\sqrt {Variance} }}} \right)}^{3} }$$

where *G*(*i, j*) is the grey scale value of each pixel in the IVUS image. *I* and *J* are the dimensions of the matrix.

#### Grey level co-occurrence matrix

Haralick [7] suggested the GLCM, which extracts features based on information on the spatial relationship between pixels. GLCM generates a co-occurrence matrix *P*(*i, j|d, θ*) from the original image by calculating the frequency of a pair of pixels with distance *d* and angle *θ* [8]. In this procedure, *i* and *j* represent the gray-scale values of two pixels. In this study, distance 1 was adapted to minimize computational time. The angle was set as 135°. By this procedure, 17 features were extracted from each window, as shown in Table 1.

#### Law’s energy measures

LEM [9, 10] extracts texture information from the original image based on the texture energy transform. To analyze the image, LEM uses the five Laws’ vectors, which are *L5*, *E5*, *S5*, *R5*, and *W5*. These vectors represents the level, edge, spot, ripple, and wave, respectively.

The Laws’ vectors are multiplied with one another, and 5 × 5 image masks are thusly generated. In this procedure, the mean values of image masks were used for the transpose matrixes, such as *E5L5* and *L5E5*. Image masks were convoluted with the window of the IVUS image, and then nine texture images were acquired as defined in Eq. (6).

6 $$\begin{array}{*{20}l} {E5L5/L5E5\;R5E5/E5R5} \\ {S5L5/L5S5\;S5S5} \\ {R5L5/L5R5\;R5S5/S5R5} \\ {S5E5/E5S5\;R5R5} \\ {E5R5} \\ \end{array}$$

Finally, the mean value of the square sum (MSS) and the mean value of the absolute sum (MAS) were calculated from texture images using Eqs. (7) and (8).

7 $$MSS = \frac{1}{I \times J}\sum\limits_{i = 1}^{I - 1} {\sum\limits_{j = 1}^{J - 1} {G(i,j)^{2} } }$$

8 $$MAS = \frac{1}{I \times J}\sum\limits_{i = 1}^{I - 1} {\sum\limits_{j = 1}^{J - 1} {\,\left| {G(i,j)} \right|} }$$

where *G*(*x, y*) is the grey scale value of each pixel in the IVUS image. In this study, 18 LEM features were extracted from each window of the IVUS image.

#### Intensity

**Fig. 1:**
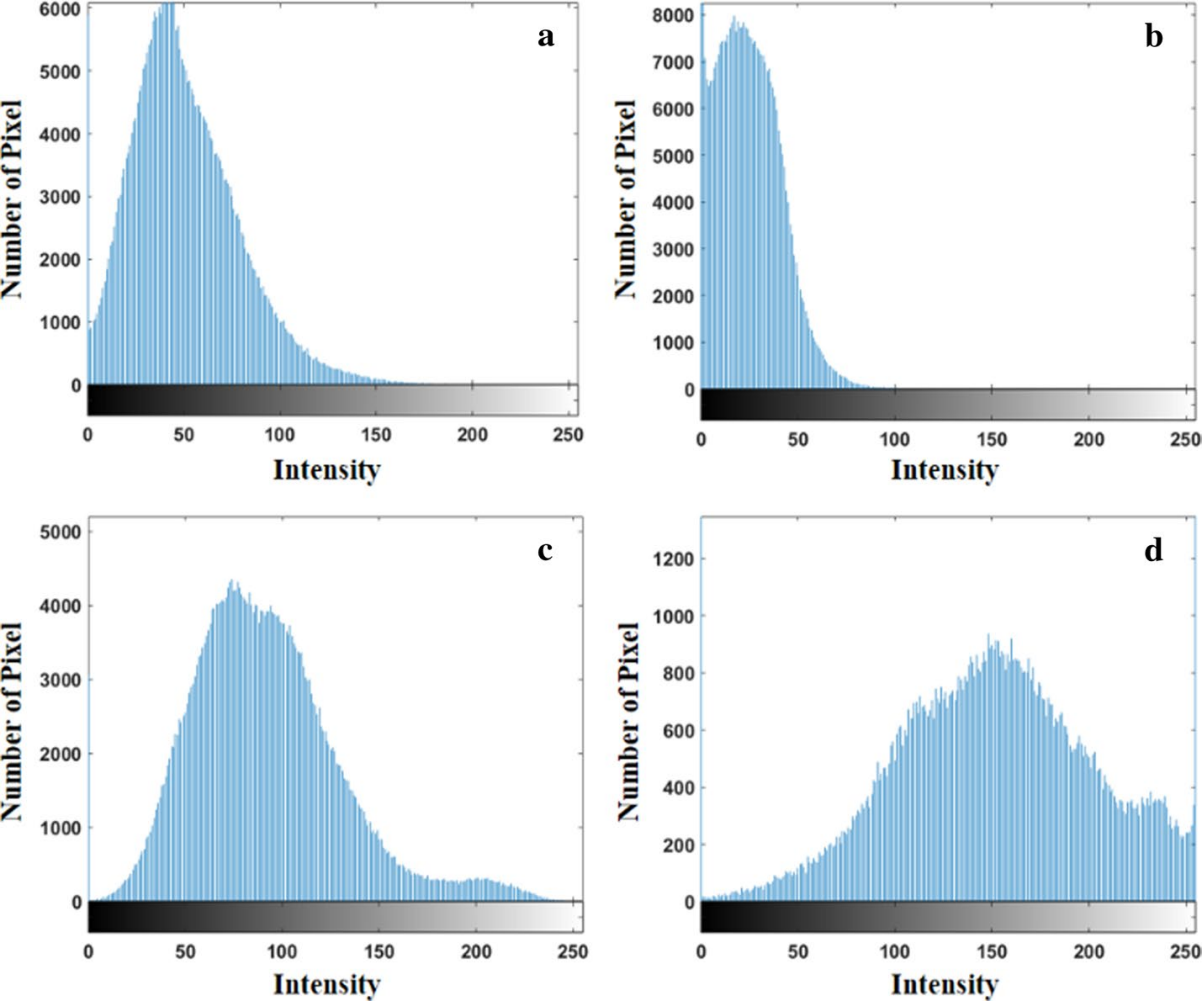
Histogram for the components of plaque (**a** fibrous tissue, **b** fibro-fatty tissue, **c** necrotic core, and **d** dense calcium)

Intensity is a simple texture feature that signifies the grey scale value of a pixel. Each plaque component commonly shows a different intensity distribution (Fig. 1). NC and DC are associated with higher intensity than are FT and FFT. In particular, DC involves the highest intensity components, because it is echogenic on ultrasound. In this study, intensity was extracted for each plaque component in order to improve classification accuracy.

#### Extended grey level run length matrix

GLRLM [11–15] extracts higher order statistical texture information. It reconstitutes the original image into a two-dimensional matrix based on the grey scale values of the pixels. Run length matrix *P*(*i, j|θ*) was calculated by counting the repeated number of grey scale value *i* with run length *j* in condition of angle *θ*. In many previous studies, five conventional GLRM features, short run emphasis, long run emphasis, grey level non-uniformity, run length non-uniformity, and run percentage, were generally used for analysis of plaque components. However, previous experimental results were not satisfactory, because they only considered the length of runs when analysing the texture information of IVUS images [1]. Therefore, in this study, the extended GLRLM features that include six additional features compared with conventional GLRLM were extracted to obtain diverse texture information from IVUS images (Table 1).

#### Local binary pattern

LBP [1, 12] analyses the binary pattern based on local structural information of the original image and detects uniform texture features. To accomplish this, LBP appoints the circular symmetric neighbourhood pixels that are at distance of radius R from the central pixel. Then, a binary digit value is obtained by subtracting the central pixel from each neighbour pixel as shown in Eq. (9). This value was passed to Eq. (10), and the basic LBP was calculated.

9 $$s(x) = \left\{ {\begin{array}{*{20}c} {1, \quad if \,\, x \ge 0} \\ {0, \quad if \,\,x < 0} \\ \end{array} } \right.$$

10 $$LBP = \sum\limits_{n = 0}^{N - 1} {s(\mathop G\nolimits_{n} - \mathop G\nolimits_{c} )\mathop 2\nolimits^{n} }$$

where *G*_*n*_ and *G*_*c*_ represent the grey scale values of the neighbour and central pixel, respectively. *N* is the number of neighbour pixels and *s*(*x*) is the thresholding function.

The rotation invariant uniform LBP (LBPriu2) that was defined by Ojala [13] was also calculated based on Eq. (11) [14]. The function U counted the number of transitions between 0 and 1 in the binary digit, as shown in Eq. (12).

11 $$LBP^{riu2} = \left\{ {\begin{array}{*{20}l} {\sum\limits_{n = 0}^{N - 1} {s(\mathop G\nolimits_{n} - \mathop G\nolimits_{c} ), \quad if \,\,U(LBP) \le 2} } \\ {N + 1,\quad if \,\,U(LBP) > 3} \\ \end{array} } \right.$$

12 $$U(LBP) = \left| {s(\mathop G\nolimits_{N - 1} - \mathop G\nolimits_{c} ) - s(\mathop G\nolimits_{0} - \mathop G\nolimits_{c} )} \right| + \sum\limits_{n = 0}^{N - 1} {\left| {\mathop {s(G}\nolimits_{n} - \mathop G\nolimits_{c} ) - s(\mathop G\nolimits_{n - 1} - \mathop G\nolimits_{c} )} \right|}$$

In our study, two LBP features were extracted form a 3 × 3 window of an IVUS image through the process discussed above.

### Feature selection

Feature selection is the process that chooses the optimal feature set to improve the classification accuracy for the lesion. If the whole of features that is acquired in the process of feature extraction is used as input for the classification, computational time will be unnecessarily long. Furthermore, a large amount of the data may occur the curse of dimensionality that decreases the classification accuracy. Therefore, this study applied PCA to select optimal features.

PCA [15–17] decreases the dimensionality of the feature space and optimizes the feature set. For this purpose, PCA was used to identify the direction vector that has the greatest variance from the feature data. Then, the principle component (PC) was acquired by projecting the original data along the direction vector. PC has reduced feature value dimensionality compared to the original data, because overlapping data was removed. Through the above process, PCA reduced the mean square error variance and provided better information on each plaque component. In this study, the optimal feature set was selected from the original feature set by using PCA for tissue characterization.

### Classification

**Fig. 2:**
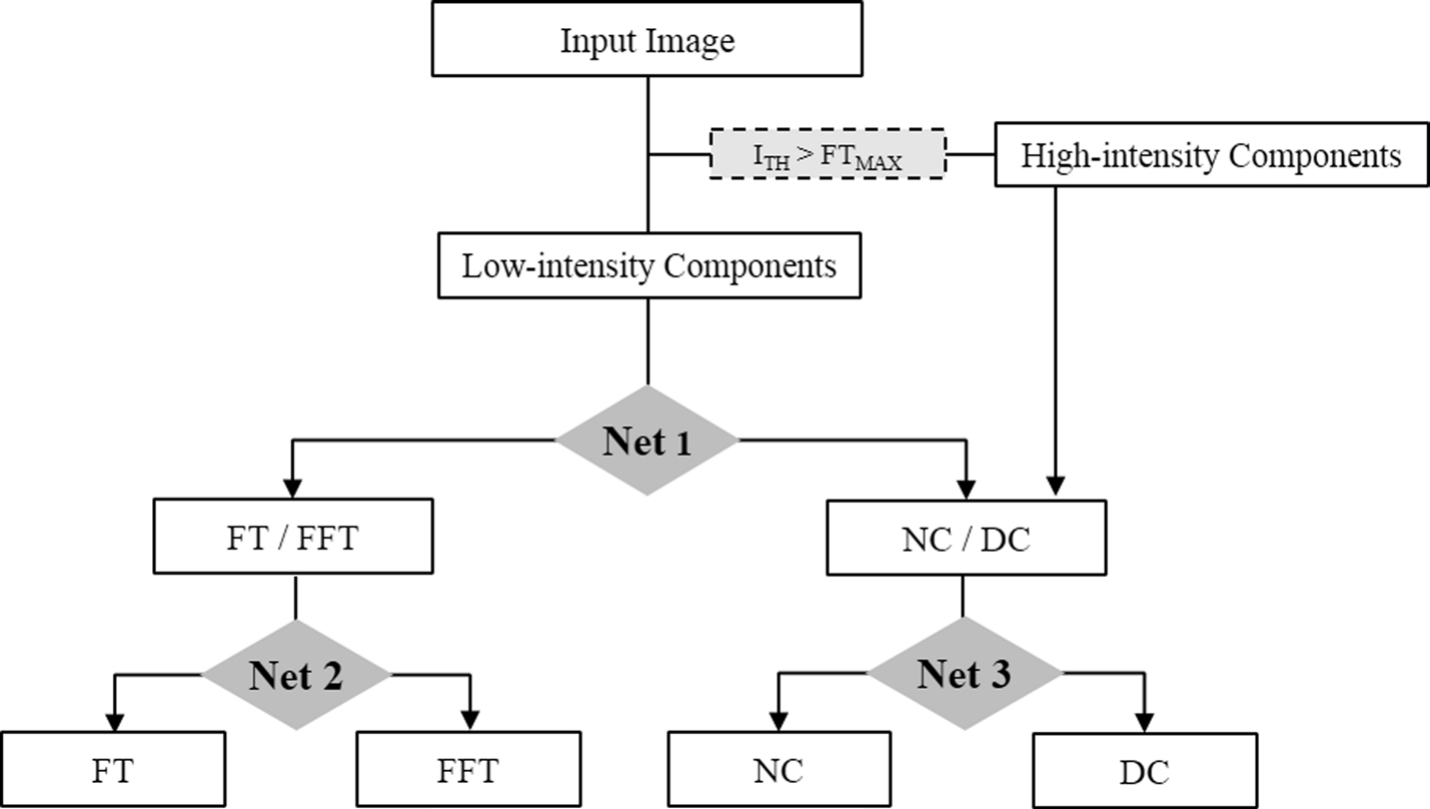
Process of the intensity-based multi-level classification

In the process of the classification, data is assigned to a pre-defined class based on the knowledge obtained during training [18]. The proposed method classified plaque components into FT, FFT, NC, or DC through the below four steps (Fig. 2).

First, the plaque components were divided into two groups based on intensity. Each plaque component shows a different intensity distribution as shown in Fig. 1. NC and DC shows higher intensity compared with FT and FFT. A key point of the proposed method is that the plaque components with higher intensity values than FT are involved in NC or DC. Therefore, the maximum value of FT was appointed as a threshold, and the plaque components were divided into low- or high-intensity components based on this threshold. High-intensity components consist of NC and DC, and the remainder consist of components with lower intensity value than the designated threshold. Next, low-intensity components were divided into the FT/FFT or NC/DC groups using net 1. Then, the net 2 classified the FT/FFT group into FT or FFT. Finally, the remaining and high-intensity components were differentiated into NC or DC via net 3. Each net was trained based on the feature set that was selected by PCA. A random forest operated by the construction of multiple decision trees was used as a classifier. To prevent over-fitting of the classifier and decrease computational time, depth and the number of trees were set at 10 and 100, respectively.

### Performance evaluation

To evaluate the performance of the proposed method, sensitivity, specificity, accuracy, and receiver operating characteristic (ROC) curve were applied. Sensitivity, specificity, and accuracy assess the ability of a classification model based on the error rate that includes true positive (TP), true negative (TN), false positive (FP), and false negative (FN). Each evaluation index was calculated using Eqs. (13) to (15).

13 $$Sensitivity = \frac{TP}{TP + FN}$$

14 $$Specificity = \frac{TN}{FP + TN}$$

15 $$Accuracy = \frac{TP + TN}{TP + TN + FN + FP}$$

Sensitivity indicates the percentage of data that were correctly classified as positive, while specificity indicates the percentage of data that were correctly classified as negative. Accuracy measures the ability of the proposed method to identify total data [19].

ROC curve is a graphical plot that represents 1-specificity and sensitivity on the x and y axes [19]. In this study, area under the curve (AUC) was additionally used as an index to evaluate the performance of the proposed method. AUC represents the value between zero and one, and a higher AUC value means a higher classification performance.

## Results

### Selected feature sets for each net by PCA

Different feature sets were optimized for the three nets by using PCA method. For net 1, 21 features were selected as the optimal feature set; they consist of the FOS, GLCM, and LEM (Table 2). Intensity was selected for nets 2 and 3 in addition to those three kinds of features. As shown in Tables 3 and 4, the feature set of net 2 includes 15 components, while 18 components were selected for net 3. GLRLM and LBP was not chosen for all nets. On the other hand, 10 features were common to the three nets, and they consist of mean, autocorrelation, variance, sum average, MSS E5L5/L5E5, MSS S5L5/L5S5, MSS R5L5/L5R5, MAS E5L5/L5E5, MAS S5L5/L5S5, and MAS R5L5/L5R5. For all three nets, most LEM features were selected as the optimal value. In particular, 15 features of 18 LEM features were chosen for net 1.

**Table 2:** Selected feature set for net 1

Feature set	Feature	Feature set	Feature
FOS	Mean Variance Standard deviation	LEM	MSS S5E5/E5S5 MSS R5E5/E5R5 MSS S5S5 MSS R5S5/S5R5 MAS E5L5/L5E5 MAS S5L5/L5S5 MAS R5L5/L5R5 MAS E5E5 MAS S5E5/E5S5 MAS R5E5/E5R5 MAS S5S5
GLCM	Autocorrelation Variance Sum average		
LEM	MSS E5L5/L5E5 MSS S5L5/L5S5 MSS R5L5/L5R5 MSS E5E5

**Table 3:** Selected feature set for net 2

Feature set	Feature	Feature set	Feature
FOS	Mean	LEM	MSS R5L5/L5R5 MSS R5R5 MAS E5L5/L5E5 MAS S5L5/L5S5 MAS R5L5/L5R5 MAS R5S5/S5R5 MAS R5R5
GLCM	Autocorrelation Variance Sum average Sum variance		
LEM	MSS E5L5/L5E5 MSS S5L5/L5S5		
		Intensity	Intensity

**Table 4:** Selected feature set for net 3

Feature set	Feature	Feature set	Feature
FOS	Mean	LEM	MSS R5S5/S5R5 MSS R5R5 MAS E5L5/L5E5 MAS S5L5/L5S5 MAS R5L5/L5R5 MAS S5S5 MAS R5S5/S5R5 MAS R5R5
GLCM	Autocorrelation Variance Sum average Sum variance		
LEM	MSS E5L5/L5E5 MSS S5L5/L5S5 MSS R5L5/L5R5 MSS S5E5/E5S5		
		Intensity	Intensity

### Classification results of proposed method for each net

Table 5 shows the results of the tissue classification for each net produced by using proposed method. Classification accuracy was in the order of net 1 > net 3 > net 2. Net 2 showed relatively low classification results, especially for specificity, which was identified as 59.6%. On the other hand, in net 1, sensitivity, specificity, and accuracy were higher than 80.0%. The proposed method also presented a high AUC of 0.845 for net 1. Furthermore, net 3 showed a relatively high classification accuracy, 77.2%.

**Table 5:** Classification results of the proposed method

Net	Sensitivity (%)	Specificity (%)	Accuracy (%)	AUC
Net 1	82.0	87.1	85.1	0.845
Net 2	81.2	59.6	71.9	0.704
Net 3	80.6	75.9	77.2	0.783

### Comparison of the classification results according to different feature selection methods

To evaluate the significance of the feature set that was selected using PCA, the classification results were compared with a genetic algorithm (GA). GA selected 25, 23, and 25 feature components for nets 1, 2, and 3, respectively. PCA showed higher classification ability than GA for all nets as shown in Tables 6, 7, and 8. According to applying the GA, the accuracy and AUC slightly decreased in net 1 and 2 than was the case without feature selection. On the other hand, PCA improved the classification results for all nets. Especially, classification accuracy of net 3 increased from 76.7 to 77.2%.

**Table 6:** Classification results of net 1 according to different feature selection methods

Selection method	Sensitivity (%)	Specificity (%)	Accuracy (%)	AUC
w/o selection	82.0	86.9	85.0	0.845
GA	81.9	86.9	84.9	0.844
PCA	82.0	87.1	85.1	0.845

**Table 7:** Classification results of net 2 according to different feature selection methods

Selection method	Sensitivity (%)	Specificity (%)	Accuracy (%)	AUC
w/o selection	80.8	60.1	71.9	0.705
GA	80.8	59.7	71.7	0.703
PCA	81.2	59.6	71.9	0.704

**Table 8:** Classification results of net 3 according to different feature selection methods

Selection method	Sensitivity (%)	Specificity (%)	Accuracy (%)	AUC
w/o selection	80.4	75.2	76.7	0.778
GA	80.2	75.8	76.6	0.780
PCA	80.6	75.9	77.2	0.783

### Comparison of the classification results according to different classifiers

Tables 9, 10, and 11 show the classification results of the coronary plaque components according to different classifiers. The proposed method showed slightly lower accuracy in net 3 compared with a dropout neural network (DNN). However, the proposed method achieved much higher classification performance in net 2 than the DNN. In particular, the proposed method showed a significantly high sensitivity of 81.2%, while a very low sensitivity of 2.1% was observed when using the DNN. It also represented higher accuracy, of about 27.8%, than did the DNN for net 2. Furthermore, the proposed method showed slightly higher accuracy and AUC for all nets in comparison with a feed forward neural network (FFNN).

**Table 9:** Classification results of net 1 according to different classifiers

Classification method	Sensitivity (%)	Specificity (%)	Accuracy (%)	AUC
DNN	79.4	86.5	84.0	0.829
FFNN	78.0	87.5	84.2	0.827
Proposed method	82.0	87.1	85.1	0.845

**Table 10:** Classification results of net 2 according to different classifiers

Classification method	Sensitivity (%)	Specificity (%)	Accuracy (%)	AUC
DNN	2.1	99.9	44.1	0.510
FFNN	81.3	57.9	71.2	0.696
Proposed method	81.2	59.6	71.9	0.704

**Table 11:** Classification results of net 3 according to different classifiers

Classification method	Sensitivity (%)	Specificity (%)	Accuracy (%)	AUC
DNN	79.2	79.9	77.5	0.780
FFNN	78.5	76.0	76.7	0.772
Proposed method	80.6	75.9	77.2	0.783

## Discussion

In order to differentiate the coronary plaque components in an IVUS image, VH, which analyzes an image based on the RF signal, has been widely applied. However, it has limitations in longitudinal resolution, and it requires specific software to acquire the RF signal data. In this study, FOS, GLRLM, LEM, intensity, extended GLRLM, and LBP features were extracted, and they were optimized using PCA. Then, an intensity-based multi-level classification model was used to classify the coronary plaque components into FT, FFT, NC, and DC based on the texture information in the IVUS image.

In the present study, three different feature sets were individually selected by applying PCA to each net. The results showed that a large number of LEM features were selected for all nets. In particular, LEM features accounted for more than 70% of 21 selected features in net 1. LEM provides diverse texture information that includes level, edge, spot, ripple, and wave for each window. Therefore, many LEM features were considered as the most significant features for classification of coronary plaque components. If an MSS feature was selected as part of the optimal feature set, the MAS feature of that image mask was also chosen. This is because the MSS and MAS have a high correlation to the same image mask. Also, PCA commonly selected 10 features in all nets, which are the mean, autocorrelation, variance, sum average, MSS E5L5/L5E5, MSS S5L5/L5S5, MSS R5L5/L5R5, MAS E5L5/L5E5, MAS S5L5/L5S5, and MAS R5L5/L5R5. Thus, 10 features were identified as essential indicators for classification of plaque components. However, GLRLM and LBP were not selected for all nets. These two types of features were affected by noise within the IVUS image. Noise may occur in the process of receiving, coding, and transmission of IVUS image [20]. Binary data generated in the process of extracting LBP features is sensitive to noise [21]. GLRLM is also extremely susceptible to noise in IVUS images [22]. For this reason, GLRLM and LBP did not show high significance in the analysis of IVUS images.

The proposed method classified the coronary plaque components based on the selected optimal feature sets, and the results showed a high accuracy (85.1%) and AUC (0.845) for net 1. This is because of the obvious difference between the FT/FFT and NC/DC groups, as shown in Fig. 1. On the other hand, the grey scale based classification method has some technical limitations in differentiating between FT and FFT, because both are medium echo reflective and show similar characteristic in IVUS images [23, 24]. Therefore, the classification results presented relatively low accuracy for net 2 (71.9%). Nevertheless, it showed a comparatively high sensitivity of 81.2%, which indicates that the proposed method has a high ability to classify FFT. Therefore, it is expected to improve overall classification accuracy by increasing ability to accurately identify for the FT.

By comparing classification accuracy according to different feature selection methods, PCA showed the highest performance in terms of computational load. Using all features, which consisted of 54 components, resulted in longest test time for the proposed classification model, 673 s. It was slightly decreased to 624 s by applying GA. By comparison, PCA greatly improved test time to 189 s, which shows its high effectiveness in terms of computational time.

The proposed method showed higher classification performance than did other classifiers including FFNN and DNN. In particular, the proposed method showed a relatively high accuracy of 71.9% in net 2, while the lowest accuracy, 44.1%, was identified for DNN. These results occurred because of the inherit characteristics of the random forest. The random forest presents high classification accuracy for data that includes noise, because it consists of multiple decision trees that have different properties. For this reason, the proposed method showed high classification ability for FT and FFT, which are difficult to differentiate because of their similar grey scale, compared with other classifiers.

This study has two main limitations. First, it did not acquire significant classification results compared with VH. It is considered that the results were affected by the quality of IVUS images used in the study. The proposed method is greatly influenced by image resolution, because it classifies plaque components based on a grey scale of image. Therefore, classification accuracy may be improved by using IVUS images with higher frequency than 20 MHz. Furthermore, the proposed method showed relatively low classification performance for high-intensity components. This seems to be affected by the amount of NC data that is included in the high-intensity components. High-intensity components are mostly composed of DC, and NC is included in relatively low proportions. The classifier may be not properly trained to identify NC, which shows high-intensity values owing to the low amount of NC data. Therefore, classification accuracy may be improved by supplementing the data on NC.

## Conclusions

This study proposed a novel intensity-based multi-level classification model to classify coronary plaque components in IVUS images. The proposed method selected 10 features that included FOS, GLCM, and LEM as key indicators for the characterization of plaque components. Quantitative results indicated that the proposed method showed significantly high classification accuracy for all tissue types. Net 1, 2, and 3 classifiers revealed classification accuracies of 85.1%, 71.9%, and 77.2%, respectively, and the areas under the curve were 0.845, 0.704, and 0.783. In particular, the proposed method achieved relatively high sensitivity (82.0%) and specificity (87.1%) for differentiating between FT/FFT and NC/DC groups. These results confirmed the clinical applicability of the proposed approach for IVUS-based tissue characterization. To improve classification accuracy, future studies should include additional experiments with a greater amount of NC data. Tissue characterization of 45 MHz IVUS images needs to be validated with various textural feature sets. Moreover, another experiment on controlling the level of noises in the various patterns will be performed.
